# An Object-Oriented Simulator for 3D Digital Breast Tomosynthesis Imaging System

**DOI:** 10.1155/2013/250689

**Published:** 2013-11-25

**Authors:** Saeed Seyyedi, Kubra Cengiz, Mustafa Kamasak, Isa Yildirim

**Affiliations:** ^1^Department of Electrical and Electronics Engineering, Istanbul Technical University, 34469 Istanbul, Turkey; ^2^Department of Computer and Informatics Engineering, Istanbul Technical University, 34469 Istanbul, Turkey

## Abstract

Digital breast tomosynthesis (DBT) is an innovative imaging modality that provides 3D reconstructed images of breast to detect the breast cancer. Projections obtained with an X-ray source moving in a limited angle interval are used to reconstruct 3D image of breast. Several reconstruction algorithms are available for DBT imaging. Filtered back projection algorithm has traditionally been used to reconstruct images from projections. Iterative reconstruction algorithms such as algebraic reconstruction technique (ART) were later developed. Recently, compressed sensing based methods have been proposed in tomosynthesis imaging problem. We have developed an object-oriented simulator for 3D digital breast tomosynthesis (DBT) imaging system using C++ programming language. The simulator is capable of implementing different iterative and compressed sensing based reconstruction methods on 3D digital tomosynthesis data sets and phantom models. A user friendly graphical user interface (GUI) helps users to select and run the desired methods on the designed phantom models or real data sets. The simulator has been tested on a phantom study that simulates breast tomosynthesis imaging problem. Results obtained with various methods including algebraic reconstruction technique (ART) and total variation regularized reconstruction techniques (ART+TV) are presented. Reconstruction results of the methods are compared both visually and quantitatively by evaluating performances of the methods using mean structural similarity (MSSIM) values.

## 1. Introduction 

Breast cancer is one of the three most commonly diagnosed types of cancer among the women in the USA in 2012. It is also known as one of the most common causes of cancer deaths in the USA [1]. Diagnosis of this type of cancer in its early stages makes the treatment simpler and more likely to be effective. 

Digital breast tomosynthesis (DBT) is an innovative imaging modality that provides 3D reconstructed images of a patient's breast to diagnose the breast cancer [2]. Conventionally several imaging modalities such as mammography and ultrasound have been used in diagnosing breast cancers. Among those modalities, X-ray mammography has been regarded as the gold standard for diagnosis. Since X-ray mammography image is two-dimensional, it is limited by overlapping tissue structure [3]. The first study of geometric tomography by Plantes introduced the concept of conventional tomosynthesis [4]. Garrison et al. [5], Richards [6], Miller et al. [7], and Grant [8] were the first scientists who studied three-dimensional tomography. Moreover Grant in his study introduced the term “tomosynthesis” system [8]. DBT overcomes the overlapping limitation of mammography by providing slice images of the breast. DBT uses projections obtained with an X-ray source moving in a limited angle interval to reconstruct 3D image of breast.

**Figure 1:**
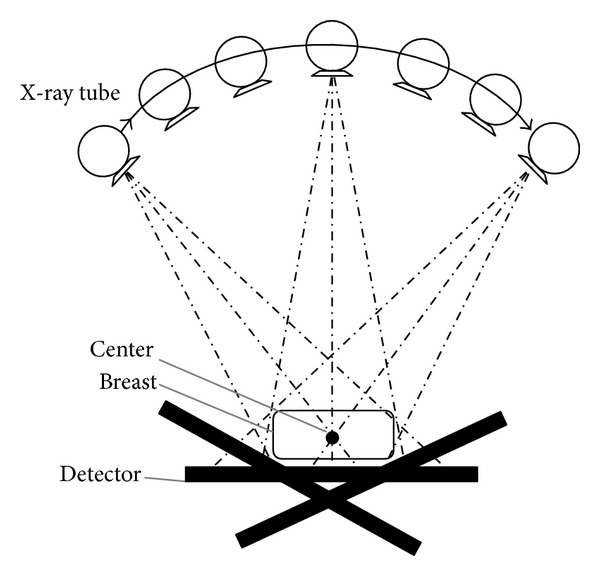
A simple schematic of digital tomosynthesis system.

A number of algorithms have been addressed to reconstruct the images. Algebraic reconstruction technique (ART) was developed by the Polish mathematician Kaczmarz in 1937 [9]. Recently it has been proven that a sparse image can be reconstructed from an undersampled data set via total variation (TV) method [10, 11].  Figure 1 schematically illustrates a digital tomosynthesis system and its three main parts: X-ray source, object, and detector. As shown in this figure, the X-ray source rotates around the breast in the step-and-shoot (SAS) mode and makes exposure after a complete stop at each position. The breast is fixed using a set of pedals to avoid the movement during the scan time and the detector is capable of high frame rate and has exceptional detective quantum efficiency (DQE), making it well suited to rapid acquisition of a large number of low-dose projection images [2, 3]. 

DBT has the potential to improve the sensitivity in the detection of breast cancer due to reduced overlap of breast tissues, which enables earlier detection. It also significantly improves the specificity; with the 3D data available, a 3D analysis of the distribution of microcalcifications, or a 3D analysis concerning shape, determining margins and size of lesions might be easier [3, 12].

Several simulators have been implemented to simulate the reconstruction algorithms. In 1970, SNARK was developed by Richard Gordon to evaluate different reconstruction algorithms. Later, different versions of SNARK were developed to simulate CT and PET systems [13]. In 2010, Hansen et al. developed AIR Tools package for 2D algebraic reconstruction techniques on MATLAB [14]. Both packages were implemented only for 2D models.

In this study we introduce a simulator for 3D breast tomosynthesis imaging system using C++ programming language. There are other limited view angle imaging simulators available such as AIR tools. However, the former simulation software was typically developed using MATLAB scripts for 2D data. Our simulator is specially designed to simulate a DBT system that takes projections of an arbitrary phantom and reconstructs it using the acquired images from projections by applying one of the implemented reconstruction methods in the simulator that is chosen by the user. It is also able to run a set of newly proposed reconstruction methods with total variation (TV) regularization algorithms and produce the results such as the image of the layer of interest, contrast to noise (CNR), root mean square error (RMSE), and structural similarity (SSIM) diagrams.

In this paper we briefly describe the reconstruction techniques used in the simulator and then the characteristics of the simulator will be provided. The results obtained from the simulator on a designed sample model are given. 

## 2. Methods

### 2.1. Arithmetic Reconstruction Technique (ART)

The algebraic reconstruction technique (ART) is an iterative image reconstruction with a long history and rich literature. First of all it was designed by Kaczmarz in 1937 [9], and it was independently used by Gordon et al. in image reconstruction [13]. ART is a reconstruction algorithm that uses a set of projections to reconstruct the desired object [15, 16].

The term ray sum takes the place of the line integral in transform-based methods. The ray sum, *y*_*i*_, measured with the *i*th ray, is expressed as

(1) $$\begin{array}{c} \sum_{j=1}^{N}a_{ij}x_{j}=y_{i},\;\begin{array}{c} i=1,2,\ldots ,M\\ j=1,2,\ldots ,N, \end{array}\;\;\;\; \end{array}$$

where *a*_*ij*_ is the weighting parameter which stands for the influence of *j*th cell on the *i*th ray line integral, *x*_*j*_ is the constant intensity value of the *j*th cell, *N* is the total number of cells, and *M* is the total number of rays. Convention matrix inversion methods mentioned above would be useful to solve (1) if *M* and *N* are small and the problem is well posed. Iterative methods are introduced for ill-posed inversion problems with large values of *N* and *M*. Expanded form of (1) can be written as

(2) a11x1+a12x2+a13x3+⋯+a1NxN=y1a21x1+a22x2+a23x3+⋯+a2NxN=y2⋮aM1x1+aM2x2+aM3x3+⋯+axN=yM.

If there is a unique solution to (2), then the intersections of the planes to be defined by these equations are a single point in *N* dimensional space. Finding the solution via subsequent projections is known as the Kaczmarz method which forms the basis of ART. The implementation procedure starts with an initial guess, ${\vec{x}}^{\left(0\right)}$ at the solution, and ${\vec{x}}^{\left(0\right)}$is projected on the first plane in (2) giving ${\vec{x}}^{\left(1\right)}$. Then ${\vec{x}}^{\left(1\right)}$ is projected on the second plane giving ${\vec{x}}^{\left(2\right)}$, thus the initial guess is updated so on. This procedure can be formulated as projection of ${\vec{x}}^{\left(i-1\right)}$ on ith plane yields ${\vec{x}}^{\left(i\right)}$:

(3) xj(i+1)=xj(i)+(yi−∑k=1Naik·xk(i))∑k=1Naik2aij,   j=1,2,…,Ni=1,2,…,M.

Equation (3) states that the previous intensity values of the estimated image, ${\vec{x}}^{(i-1),}\text{s}$, are updated by adding an error parameter Δ*x*_*j*_^(*i*)^ which is the difference between measured ray sum, *y*_*i*_, and the computed ray sum, ∑_*k*=1_^*N*^ *a*_*ik*_ · *x*_*k*_^(*i*−1)^, normalized by ∑_*k*=1_^*N*^ *a*_*ik*_^2^. This process is repeated until all the projections are considered and all the pixel values converge to a solution [13, 16].

### 2.2. Simultaneous Arithmetic Reconstruction Technique (SART)

ART method was the first iterative algorithm used in CT [13]. In 1984, the simultaneous algebraic reconstruction technique (SART) was proposed with major alterations in the ART [17, 18]. SART, as described by Andersen and Kak (1984), is given by

(4) $$\begin{array}{c} x_{j}^{\left(k+1\right)}=x_{j}^{k}+\frac{\omega }{\sum_{i}^{}a_{ij}}\sum_{i}^{\;}\frac{a_{ij}\left(y_{i}-\sum_{m}^{}a_{im}x_{m}^{\left(k\right)}\right)}{\sum_{k}^{}a_{ik}}, \end{array}$$

where 0 < *ω* < 2 represents relaxation parameter; for iterations *k* = 0, 1,…, *k* we set *ω* to 1 for our simulation. Although larger values may speed up convergence, if the value is too large, too much weight is given to the last projection, which prevents convergence. Smaller values cause the algorithm to converge slowly, which is not acceptable for real-time applications and systems with a huge number of pixels [19].

### 2.3. Compressed Sensing (CS)

Compressed sensing (CS) image reconstruction is used to reconstruct a sparse image by minimizing the *l*1 norm of the sparse image. There are some significant factors in original CS method to be considered: (1) the image must be sparse; (2) reconstruction of the image must be done using a nonlinear method; and (3) the standard linear reconstruction method should generate incoherent view aliasing artifacts by applying the sparsifying transform in (7) [10, 11]. The image can be sparsified using sparsifying transform (Ψ) which is a linear transform operator and is used to transform nonsparse version of image *X* to the sparsified version. Equation (5) shows the constrained minimization problem which CS image reconstruction theory tries to solve iteratively:

(5) $$\begin{array}{c} \;\min{||\Psi X||}_{1} \end{array}$$

(6) $$\begin{array}{c} \text{s.t}.\;AX=Y. \end{array}$$

### 2.4. Prior Image Constrained Compressed Sensing (PICCS)

PICCS method considers a high quality prior image *X*_*P*_ to reconstruct the image *X* from an undersampled data set by solving the following constrained minimization problem:

(7) $$\begin{array}{c} \min\left[\alpha {||\Psi_{1}\left(X-X_{Y}\right)||}_{1}+\left(1-\alpha \right){||\Psi_{2}\left(X\right)||}_{1}\right], \end{array}$$

where *AX* = *Y* is assumed and Ψ_1_ and Ψ_2_ can be any transform like those used in CS and they can be the same or different transforms, and *α* is the regularization parameter that can be selected between 0 and 1; for *α* = 0 the PICCS algorithm is equivalent to the known CS method [10, 11]. 

The constrained minimization problem of PICCS method is numerically implemented using arithmetic reconstruction technique (ART) and the total variation (TV) regularization methods, respectively. ART is used to reconstruct the image *X* by considering the consistency condition *AX* = *Y* and TV regularization of *X* is defined as *l*1 norm of the discrete gradient of the image. Equation (8) shows the 2D TV of pixel *X*(*i*, *j*) in the image

(8) $$\begin{array}{c} {\text{TV}}_{2\text{D}}\left(X\left(i,j\right)\right)=\sum_{i,j=1}^{N}{\left|\nabla_{i,j}\left(X\left(i,j\right)\right)\right|}_{1}. \end{array}$$

The discrete gradient of image in pixel (*i*, *j*) is defined as

(9) $$\begin{array}{c} \left|\nabla_{i,j}X\left(i,j\right)\right|=\sqrt{{{\left(D_{x}X\right)}^{2}+{\left(D_{y}X\right)}^{2}}^{\;}}, \end{array}$$

where *X*(*i*, *j*) is the intensity value at pixel (*i*, *j*), *D*_*x*_ *X* = *X*(*i*, *j*) − *X*(*i* + 1, *j*), and *D*_*y*_ *X* = *X*(*i*, *j*) − *X*(*i*, *j* + 1).

TV regularization can be assumed in 3D objects where it shows better performance in the *z*-axis neighborhood or axial direction of the object; (10) shows the 3D TV of the voxel *X*(*i*, *j*, *k*) in 3D object

(10) $$\begin{array}{c} \text{TV}{}_{3\text{D}}\left(X\left(i,j,k\right)\right)=\sum_{i,j,k=1}^{N}{\left|\nabla_{i,j,k}\left(X\left(i,j,k\right)\right)\right|}_{1}. \end{array}$$

The discrete gradient of image in voxel (*i*, *j*, *k*) is shown in

(11) $$\begin{array}{c} \left|\nabla_{i,j,k}X\left(i,j,k\right)\right|=\sqrt{{\left(D_{x}X\right)}^{2}+{\left(D_{y}X\right)}^{2}+{{\left(D_{z}X\right)}^{2}}^{\;}}, \end{array}$$

where *X*(*i*, *j*, *k*) is the intensity value at voxel (*i*, *j*, *k*), *D*_*x*_ *X* = *X*(*i*, *j*, *k*) − *X*(*i* + 1, *j*, *k*), *D*_*y*_ *X* = *X*(*i*, *j*, *k*) − *X*(*i*, *j* + 1, *k*), and *D*_*z*_ *X* = *X*(*i*, *j*, *k*) − *X*(*i*, *j*, *k* + 1).

The TV method is applied after each iteration of ART method. After applying TV, the forward projection runs again. TV method can be applied to 2D or 3D data. 2D TV is applied for each layer of 3D object, but, the 3D version of TV regularization is applied to the whole of the 3D object at the end of each iteration. The pseudocode of the ART with 3D TV or SART with 3D TV implementation is shown below:

 
*X*_*P*_← Prior Image
 
*Y*_*P*_← Forward Projection of *X*_*P*_
 
*Y*← Measured Projections
 
*while* (||*Y*_*P*_ − *Y*||>*ε*)
   
*for each iteration*
     Calculate Δ*X* ^*k*^ Using ART
     Update *X*_*P*_^(*k*)^ (*X*_*P*_^(*k*+1)^ = *X*_*P*_^(*k*)^ + Δ*X*_  _^(*k*)^)
   
*end for each iteration*
   3D Total Variation Regularization
   
*Y*_*P*_← Forward Projection of *X*_*P*_
 
*end while*

## 3. Software Design and Implementation

3D tomosynthesis simulator was written in C++. An object-oriented programming language and  .Net framework was used to design the graphical user interface (GUI) of the simulator on Visual Studio.Net 2010 which was run on a personal computer with Intel Core i7 2.00 GHz processor and 6 GB RAM memory. Unlike the procedural programming languages that separate data from operations, object-oriented C++ programming language is capable of considering a collection of classes that combine data and operations on data.

**Figure 2:**
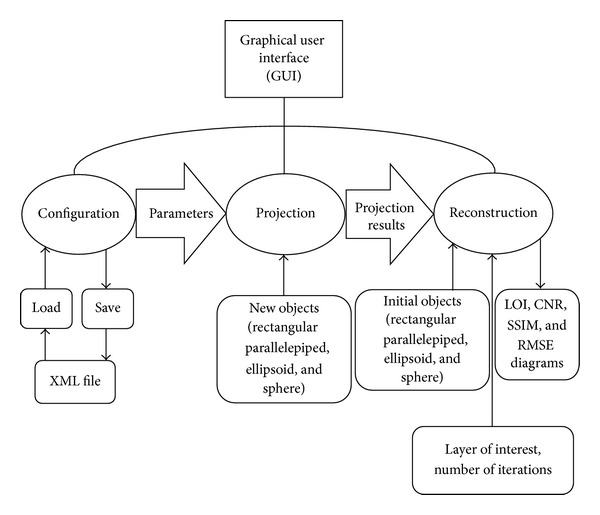
Flow diagram for DBT simulator.

Figure 2 shows the flow diagram of the simulator where three main parts of the simulator and related operators are shown. As shown in this figure, the simulator consists of three main classes: configuration (parameters), projection, and reconstruction classes. The first class includes the parameters of all the system parts and functions to read/write data from XML files. Three main parts of DBT system are defined as three different subclasses which refer to X-ray source, phantom, and detector. The projection class includes methods to receive the system parameters from the configuration part and to find the projection images of a particular phantom which could be used by the reconstruction class to run different reconstruction methods. The simulator includes a graphical user interface (GUI) which facilitates design and editing of a phantom, executes the projection and reconstruction method, and saves, the results.

**Figure 3:**
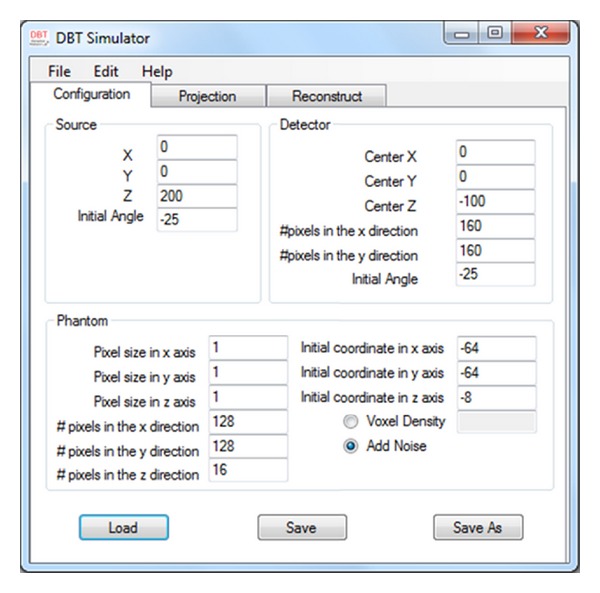
Simulator's configuration part; user inserts simulator parameters manually or by loading an xml file.

Figure 3 shows the configuration interface of the simulator that allows one to insert or select the parameters of the X-ray source, phantom, and detector such as their location and dimensions manually in the specified places or by loading the xml files that include the desired data in a predefined format. One can load the xml file by pressing the load button and inserting the path for the desired file.

It is possible to choose a set of small 3D objects, such as rectangular parallelepiped, sphere, or ellipsoid for both original and initial objects to start the reconstruction procedure. 

**Figure 4:**
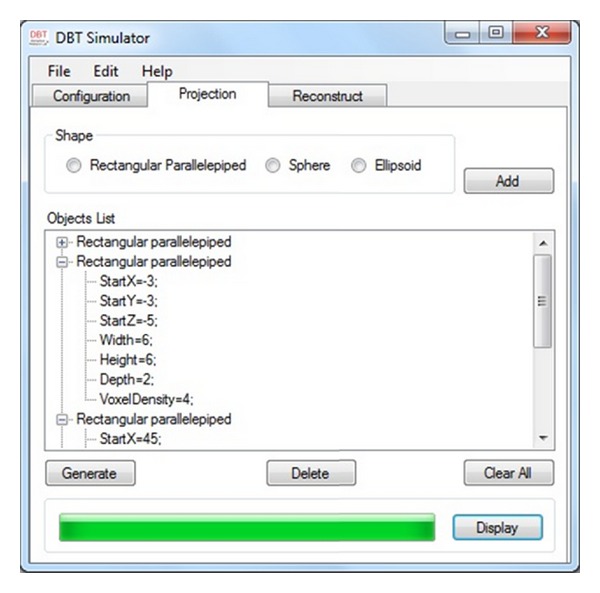
Simulator's projection part; user can insert more objects into the phantom and run the projection method.

Figure 4 shows the projection interface of the simulator; it is possible to insert and edit a set of small 3D objects such as rectangular parallelepiped, sphere, or ellipsoid in the phantom which are displayed sequentially in the object list part of the form. The user is required to insert the characteristics of each object to generate the desired phantom. After generating the phantom, the projection task can be performed to get the projection images of the phantom. The results of the projection on the detector could be displayed by choosing the desired angle of projection.

**Figure 5:**
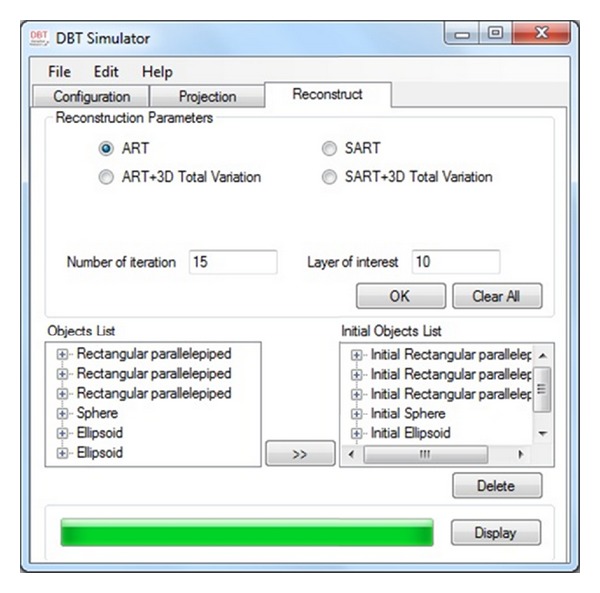
Simulator's reconstruction part; user can choose a reconstruction method, insert the layer of interest and number of iterations, and change the initial objects characteristics then run the desired method.

Figure 5 exhibits the reconstruction interface where the user can choose one of the reconstruction methods including ART, SART, ART with 3D TV, and SART with 3D TV, and can insert the number of iterations (NOI) of the iterative method with the layer of interest (LOI) number of the 3D phantom. It is also possible to revise the initial object list for the reconstruction. After running the chosen method the reconstruction results such as the image of all layers including the LOI of the object and also the structural similarity (SSIM), contrast-to-noise-ratio (CNR), and root mean square error (RMSE) diagrams will be shown as the output of the program.

A special xml file format is designed to store all of the system characteristics using the different tags such as X-ray source, detector, phantom, and reconstruction. A user can insert and edit the contents of the file and load it automatically to update the parameters of the system.

## 4. Results

**Figure 6:**
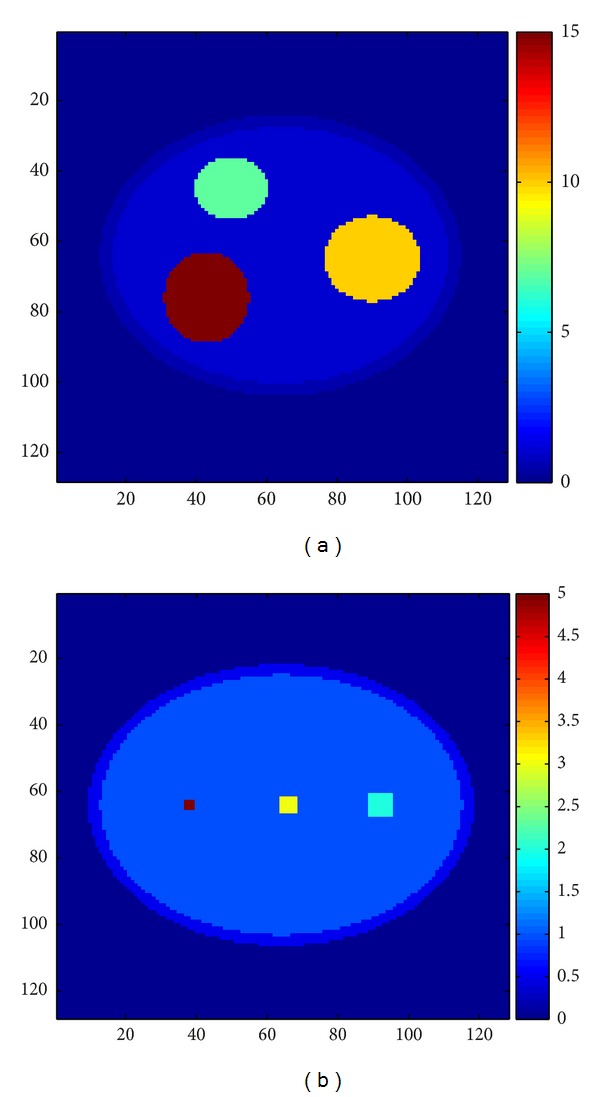
Original 3D phantom: (a) objects with higher absorption in the upper layers, (b) the LOI of the phantom.

In order to exhibit the performance of the different reconstruction methods run by the simulator, a 3D phantom model was designed with resolution of 128 × 128 voxels in 16 layers. This phantom was created to imitate the overlapping tissue problem of the breast imaging. The phantom includes some smaller objects, where objects with the low X-ray absorption are obscured by the objects with higher X-ray absorption (Figure 6). Measured projections were generated from the phantom for the range of scan angles from −25° to +25° and 11 projections. 

Parameters of the simulator and phantom are listed in Table 1.

**Figure 7:**
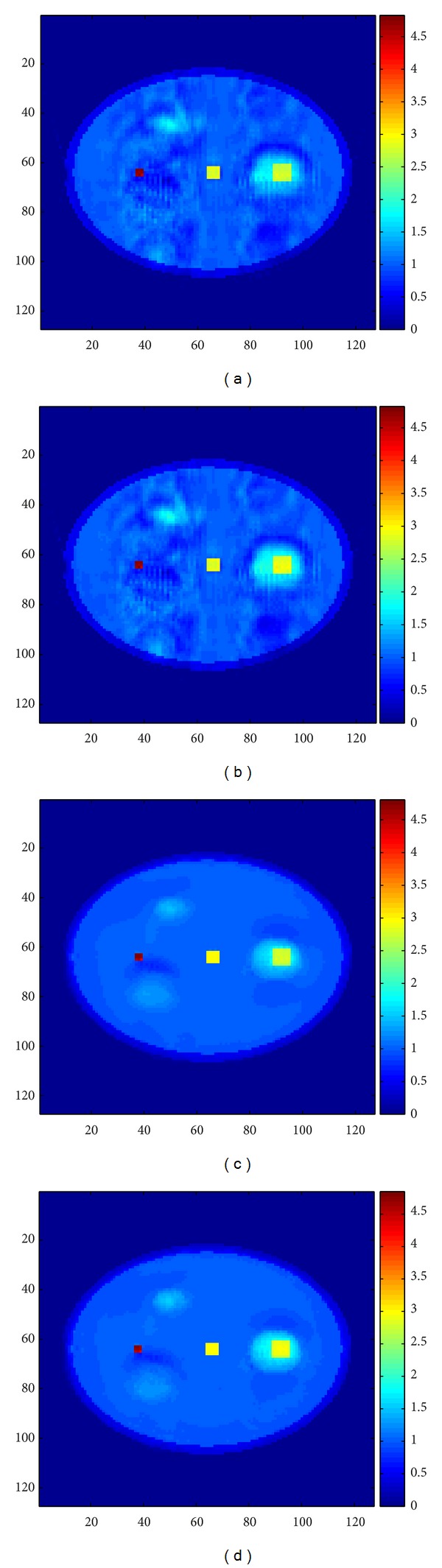
Images of the LOI of the reconstructed phantom (11th layer of the phantom): (a) reconstructed LOI image using ART method, (b) reconstructed LOI image using SART method, (c) reconstructed LOI image using ART+TV 3D method, and (d) reconstructed LOI image using SART+TV 3D method.

 Reconstructed images of the LOI for ART, SART, ART+TV 3D, and SART+TV 3D are shown in Figure 7(a) to Figure 7(d), respectively. 

One of the mostly used image quality metrics is the mean square error (MSE) because of its simplicity in calculating and clear physical meaning, but this is not a very appropriate metric to exhibit the visual quality of the images [20–22]. A number of quality assessment methods are developed that implement the characteristics of the human visual system (HVS). One of the well-known quality assessment methods is the measure of structural similarity (SSIM) that compares local patterns of pixel values which are normalized for amount of luminance and contrast [23].

The SSIM index is shown:

(12) $$\begin{array}{c} \text{SSIM}\left(x,y\right)=\frac{\left(2\mu_{x}\mu_{y}+C_{1}\right)\left(\sigma_{xy}+C_{2}\right)}{\left(\mu_{x}^{2}+\mu_{y}^{2}+C_{1}\right)\left(\sigma_{x}^{2}+\sigma_{y}^{2}+C_{2}\right)}, \end{array}$$

where *μ*_*x*_ and *μ*_*y*_ refer to the mean of the intensities of signals *x* and *y*, respectively, and *σ*_*x*_ and *σ*_*y*_ are the standard deviation of them. *C*_1_ and *C*_2_ are given:

(13) $$\begin{array}{c} C_{m}={\left(K_{m}L\right)}^{2},\;m=1,2, \end{array}$$

where *L* is the dynamic range of the pixel values and *K*_*m*_ ≪ 1 for *k* = 1,2 are small constants. 

Practically we need a single overall quality measure of the entire image. In this study we used a mean SSIM (MSSIM) index to evaluate the overall image quality:

(14) $$\begin{array}{c} \text{MSSIM}\left(X,Y\right)=\frac{1}{M}\sum_{j=1}^{T}\text{SSIM}\left(x_{j}+y_{j}\right), \end{array}$$

where *X* and *Y* refer to original and reconstructed images, respectively; *x*_*j*_ and *y*_*j*_ are the image contents at the *j*th local window and *T* is the number of local windows of the image. 

**Figure 8:**
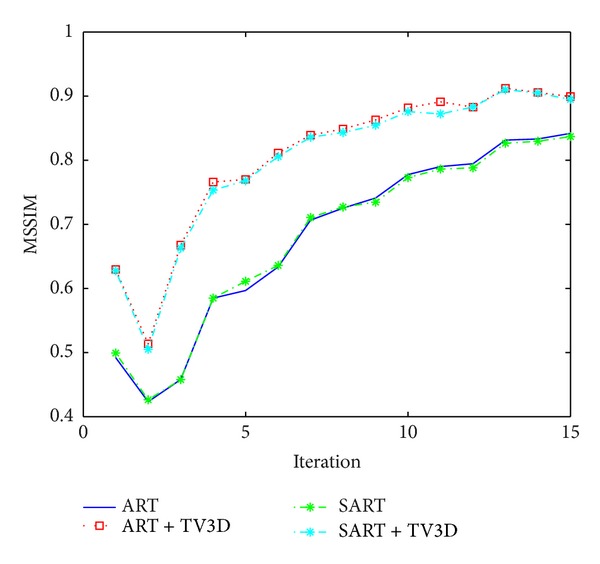
MSSIM value comparison of ART, ART+3D TV, SART, and SART+3D TV for the layer of interest.

MSSIM indexes of the reconstruction methods tested with the simulator are given in Figure 8. Compressed sensed methods implemented as ART+TV 3D and SART+TV 3D provided improved results compared with the results of ART and SART while ART and SART performed similarly. 

The time needed to perform a simulation study depends on the complexity of the phantom and detector size. In this study the average time required to complete each iteration of ART on the proposed simulator is 128 seconds which is obtained after measuring the first fifteen iterations. The same problem takes 28800 seconds on the MATLAB which was only implemented by us for comparison.

## 5. Conclusion

A new simulator was designed for 3D DBT studies. Our simulator is capable of implementing several reconstruction techniques including recently proposed compressed sensing based methods. A user friendly graphical user interface (GUI) helps users to select and run the desired methods on the designed phantom models or real data sets. The simulator was implemented for 3D limited view angle imaging problems using C++ programming language whereas the former simulation software was typically developed using MATLAB scripts for 2D data. We tested the simulator by running different reconstruction methods with a specific 3D phantom model which was created to imitate the overlapping tissue problem of the breast imaging. We also compared the methods in the simulator by demonstrating reconstructed images of LOI and evaluating their performances using RMSE and MSSIM metrics. The simulator can be extended by including new reconstruction methods.

## Figures and Tables

**Table 1:** Simulation parameters.

Parameter	Value
Source to detector distance	300 pixels
Object to detector distance	100 pixels
Scan angle	50° degrees (−25° to +25°)
Number of projections	11 projections
TV regularization parameter	0.8
Phantom size	128 × 128 × 16
Detector size	160 × 160 × 1
